# High Rates of Asymptomatic, Sub-microscopic *Plasmodium vivax* Infection and Disappearing *Plasmodium falciparum* Malaria in an Area of Low Transmission in Solomon Islands

**DOI:** 10.1371/journal.pntd.0003758

**Published:** 2015-05-21

**Authors:** Andreea Waltmann, Andrew W. Darcy, Ivor Harris, Cristian Koepfli, John Lodo, Ventis Vahi, David Piziki, G. Dennis Shanks, Alyssa E. Barry, Maxine Whittaker, James W. Kazura, Ivo Mueller

**Affiliations:** 1 The Walter & Eliza Hall Institute, Melbourne, Australia; 2 University of Melbourne, Melbourne, Australia; 3 National Health Training and Research Institute, Ministry of Health, Honiara, Solomon Islands; 4 Australian Army Malaria Institute, Brisbane, Australia; 5 National Vector Borne Disease Control Program, Ministry of Health, Honiara, Solomon Islands; 6 School of Population Health, University of Queensland, Brisbane, Australia; 7 Case Western Reserve University, Cleveland, Ohio, United States of America; 8 Barcelona Centre for International Health Research (CRESIB), Barcelona, Spain; University of Sao Paulo, BRAZIL

## Abstract

**Introduction:**

Solomon Islands is intensifying national efforts to achieve malaria elimination. A long history of indoor spraying with residual insecticides, combined recently with distribution of long lasting insecticidal nets and artemether-lumefantrine therapy, has been implemented in Solomon Islands. The impact of these interventions on local endemicity of *Plasmodium* spp. is unknown.

**Methods:**

In 2012, a cross-sectional survey of 3501 residents of all ages was conducted in Ngella, Central Islands Province, Solomon Islands. Prevalence of *Plasmodium falciparum*, *P*. *vivax*, *P*. *ovale* and *P*. *malariae* was assessed by quantitative PCR (qPCR) and light microscopy (LM). Presence of gametocytes was determined by reverse transcription quantitative PCR (RT-qPCR).

**Results:**

By qPCR, 468 *Plasmodium* spp. infections were detected (prevalence = 13.4%; 463 *P*. *vivax*, five mixed *P*. *falciparum/P*. *vivax*, no *P*. *ovale* or *P*. *malariae*) versus 130 by LM (prevalence = 3.7%; 126 *P*. *vivax*, three *P*. *falciparum* and one *P*. *falciparum/P*. *vivax*). The prevalence of *P*. *vivax* infection varied significantly among villages (range 3.0–38.5%, *p*<0.001) and across age groups (5.3–25.9%, *p*<0.001). Of 468 *P*. *vivax* infections, 72.9% were sub-microscopic, 84.5% afebrile and 60.0% were both sub-microscopic and afebrile. Local residency, low education level of the household head and living in a household with at least one other *P*. *vivax* infected individual increased the risk of *P*. *vivax* infection. Overall, 23.5% of *P*. *vivax* infections had concurrent gametocytaemia. Of all *P*. *vivax* positive samples, 29.2% were polyclonal by MS16 and *msp1*F3 genotyping. All five *P*. *falciparum* infections were detected in residents of the same village, carried the same *msp2* allele and four were positive for *P*. *falciparum* gametocytes.

**Conclusion:**

*P*. *vivax* infection remains endemic in Ngella, with the majority of cases afebrile and below the detection limit of LM. *P*. *falciparum* has nearly disappeared, but the risk of re-introductions and outbreaks due to travel to nearby islands with higher malaria endemicity remains.

## Author Summary

Solomon Islands, an island nation in the Southwest Pacific that has seen dramatic reductions in malaria transmission over the past 20 years, is aiming for malaria elimination. There is an increasing recognition that a substantial reservoir of asymptomatic and often sub-microscopic *Plasmodium spp*. infections exists even in low transmission settings. However, the potential role for these infections in sustaining transmission and the difference in response of the two most common malaria parasites, *P*. *vivax* and *P*. *falciparum*, to intensified control remains unclear. In May-June 2012, we therefore performed a cross-sectional survey of 3501 residents of all ages of Ngella, a low transmission area in Central Islands Province, to assess the prevalence of *P*. *vivax* and *P*. *falciparum* infection, determine the proportion of sub-microscopic and afebrile infections and evaluate whether gametocytaemic, and thus potentially infectious, individuals are present. Our survey showed a marked epidemiological contrast between *P*. *vivax* and *P*. *falciparum*. Although prevalence varied significantly among different regions of Ngella, *P*. *vivax* remains firmly endemic, with high rates of sub-microscopic, afebrile and genetically diverse infections. The presence of gametocytes among both sub-microscopic and microscopy positive, asymptomatic infections indicates that these infections contribute significantly to sustaining *P*. *vivax* transmission. *P*. *falciparum*, on the other hand, appears to be more amenable to control interventions. Only five *P*. *falciparum* infected individuals were detected, and all were residents of the same village. These infections carried the same *msp2* clone. This difference highlights the larger challenge of eliminating *P*. *vivax* compared to *P*. *falciparum* in areas where they are co-endemic. In particular, the challenge posed by the presence of a large reservoir of silent *P*. *vivax* infections will need to be addressed if control of this parasite is to be accelerated and elimination achieved.

## Introduction

Nations in the Southwest Pacific have endured considerable malaria transmission, with the highest *Plasmodium falciparum* burden outside the African continent and possibly the highest *Plasmodium vivax* transmission in the world [1]. Historically, transmission has ranged from hyperendemic areas in West Papua (Indonesia) and Papua New Guinea [2] to high and moderate transmission in Solomon Islands and Vanuatu [3], which are the southwestern boundary of global malaria transmission. Intensified control over the last 20 years has resulted in remarkable declines in malaria transmission in this region [3,4], reviving the agenda of elimination. However, it is in these countries where outstanding progress towards elimination has been made, that more knowledge is needed if the vision of malaria elimination is to be realized, such as reliable prevalence estimates, role of low-density, asymptomatic carriers and determinants of transmission maintenance.

In Solomon Islands, the incidence of clinical malaria cases diagnosed by light microscopy (LM) dropped by 90% from 442/1000 population in 1992 [5] to 44/1000 population in 2012 [6]. These drops in incidence are similar to those achieved by the Malaria Eradication Program in Solomon Islands (1970–1975) [7]. National statistics based on passive surveillance indicate that 65% of clinical malaria cases in 2012 were attributable to *P*. *falciparum*, 33% to *P*. *vivax* and 2% to mixed *P*. *falciparum*/*P*. *vivax*. Conversely, active case detection surveys indicate that *P*. *vivax* is the predominant species in the general population [6]. Current malaria transmission appears to be focal, ranging from moderate to high levels in Honiara City (96/1000) and Guadalcanal (64/1000) to very low in Temotu (10.8/1000) and Isabel provinces (1.2/1000).

Temotu and Isabel are the only two provinces in which pilot elimination agenda has been proposed to be actively pursued, having resulted in more intensive control activities and interventions including stratification, active case detection, and the earlier roll out of control activities (e.g. rapid diagnostic tests, RDTs and indoor residual spraying) than the rest of the country [3]. These provinces are also the only areas of Solomon Islands with recent surveys in which both LM and PCR-based diagnoses of *Plasmodium spp*. infections were performed [8,9]. In 2008, a parasite prevalence of 2.7% by LM was found in Temotu, with *P*. *vivax* accounting for 82.5% of infections. Only 5.5% of these infections were associated with febrile illness. Among a subset of 1,748 samples, which included LM positive, febrile and 10% of LM negative participants, an additional 63 *P*. *falciparum*, 23 *P*. *vivax* and 10 mixed *P*. *falciparum/P*. *vivax* infections were detected by PCR, indicating a 6.5% prevalence of sub-microscopic infections. Even lower levels of infection were reported in Isabel in 2009: 1 of 8,554 participants had a LM-detectable *P*. *falciparum* infection (0.01%). In a random subset of 2001 participants, PCR identified an additional 13 (0.55%) *P*. *vivax* infections.

PCR consistently detects at least twice as many infections as LM [10]. Numerous studies have confirmed that sub-microscopic infections are a common feature of malaria endemic areas, spanning all age groups and involving both *P*. *falciparum* and *P*. *vivax* [11–13]. Although these sub-microscopic infections are rarely associated with febrile illness, they have been shown to be efficient gametocyte producers [14–19] and thus constitute a source of ongoing transmission [10].

Given the lack of data from other areas of Solomon Islands, it is currently unknown whether the pattern of asymptomatic, low-density infection carriage identified in Temotu and Isabel [8,9] is unique to these elimination provinces. In addition, whereas these earlier surveys detected a large burden of sub-microscopic infections, they did not determine if these infections were also gametocytaemic and therefore did not assess their potential contribution to transmission. Therefore, we conducted in May-June 2012 a household-based, cross-sectional survey in Ngella, Central Islands Province to determine how common low-density, asymptomatic infections are in communities where transmission is mesoendemic and whether these infections are gametocyte producers and hence, potential contributors to local transmission. This survey is the first epidemiological description of malaria in Ngella since the 1970–1975 Malaria Eradication Program [7] and the only one in Solomon Islands to employ highly sensitive molecular diagnosis for the detection of both blood-stage parasites and gametocytes.

## Methods

### Ethics statement

This study was approved by The Walter and Eliza Hall Institute Human Research Ethics Committee (HREC number 12/01) and the Solomon Islands National Health Research Ethics Committee (HRC12/022). The informed consent process recognized the community and cultural values of Solomon Islands. Following consultation with and approval by community leaders, community meetings were held to explain the aims, risks and potential benefits of the study. Individual informed consent was obtained from all participants or the parent or legal guardian of children<18 years of age. At the point of collection, all samples were de-identified.

### Study site

**Fig 1:**
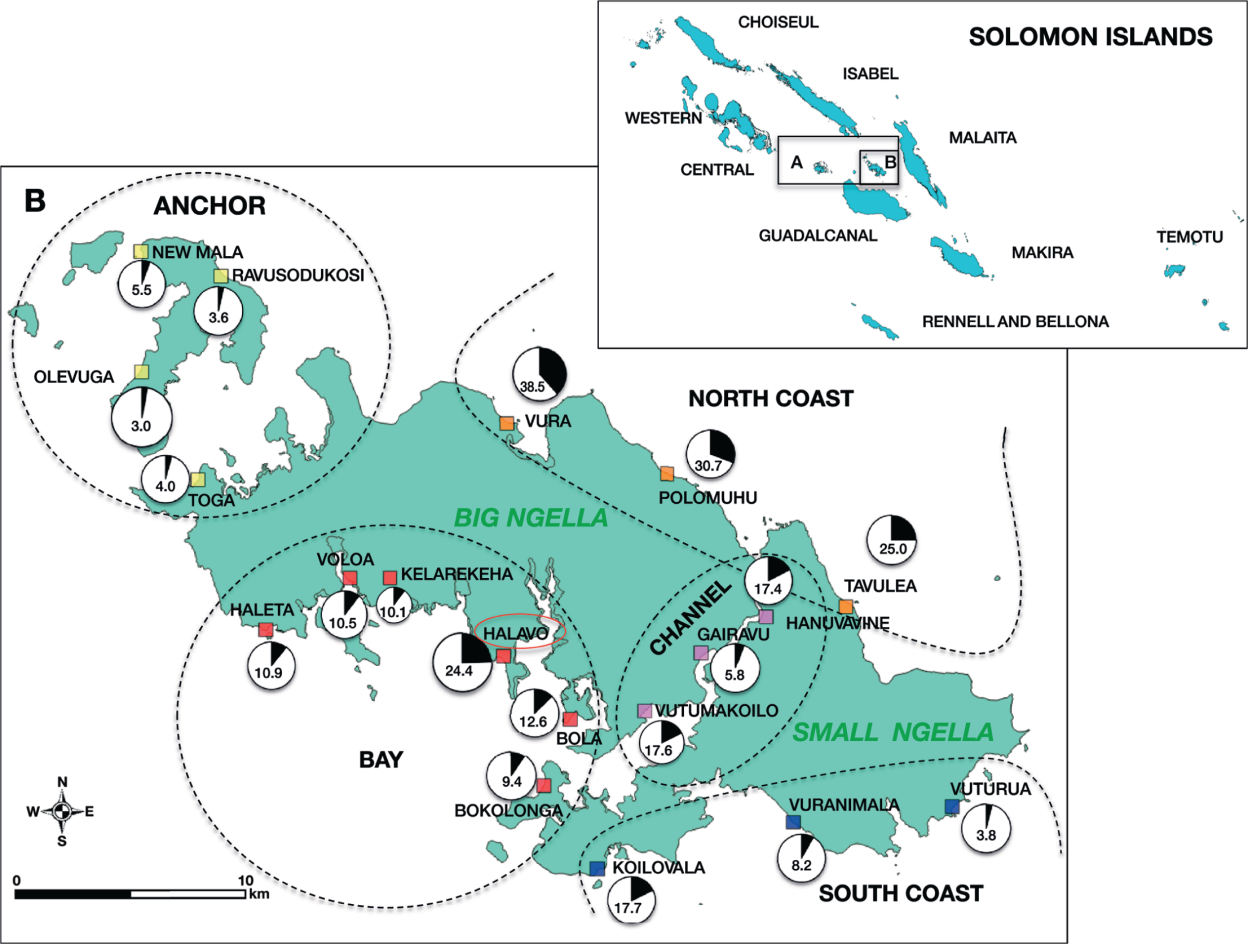
Ngella sampling sites and spatial distribution of *P*. *vivax* prevalence (qPCR). The 9 island provinces of SI are shown in the top right inset. A (inset). Central Islands Province. B (inset). Ngella. B. Ngella study catchments and prevalence. Anchor Island (catchments shown in yellow), Bay (catchments in red), Channel (catchments in purple), North Coast (catchments in orange) and South Coast (catchments in blue). The size of the prevalence pie chart reflects village sample size.

Ngella, previously known as the Florida Islands, consists of 3 islands, Anchor, Big Ngella and Small Ngella, located approximately 27 miles north of Guadalcanal and 50 miles southwest of Malaita (Fig 1). Along with Tulaghi, Savo, Russel and Buenavista Islands it forms part of the Central Islands Province (Fig 1). Despite their proximity, the three islands of Ngella have diverse geographical characteristics: Anchor Island is characterized by less dense rainforest and sandier soil. Big Ngella is heavily forested, although commercial deforestation is common, and smaller villages are encountered in the Bay area around Tulagi, the provincial capital. The more remote northern villages of Big and Small Ngella and those on the southern coast are larger. The communities of the Utuha Channel lay in an extensive mangrove system and are smaller in size. There is minimal seasonal variation in temperature and despite a northwesterly monsoon from November-April, the distinction between wet and dry season is not pronounced. The most recent census estimates 26,051 inhabitants (approximately 60% of these reside in Ngella), 49% females and a median age of 19.9 years [20]. There is significant migration between Ngella and other malaria endemic areas, in particular Honiara (Guadalcanal) and Malaita provinces. These provinces are well connected to Ngella by a popular ferry service and numerous private, unscheduled motorized boat trips. The Ngella population is serviced by a hospital in Tulagi, six rural health sub-centres and ten nurse aid posts. National malaria statistics describe Ngella as mesoendemic, with a reported Annual Parasite Index [21] of 46.1/1000 in 2012, *P*. *falciparum* being the main cause of malaria cases [6].

Overall API for Solomon Islands indicates that there were two transmission peaks in 2012 for the months of February and October. As elsewhere in the country, long lasting insecticidal nets and indoor residual spraying are the mainstay of malaria control in Ngella. Cases are diagnosed by LM or RDT and treatment with artemether-lumefantrine has been introduced nationally in 2008.

The last malaria epidemiological report of Ngella [7] described it as ‘the most malarious group in all Solomon Islands” and the “most difficult from which to clear malaria”. Malariometric surveys preceding the Malaria Eradication Program (March 1965—January 1970, unpublished World Health Organisation Field Reports (reviewed in [8]) identified a combined parasite rate of 69.6% and a spleen rate of 69.3% in the 2–9 years age group. In the same surveys, villages on the North coast had spleen rates in the 80% range and qualified for the hyperendemic classification [7], whereas the villages in the Bay area and South coast were noted to have had spleen rates in the 30–50% range [7].

### Study population and blood sample collection

A representative population sample was obtained with a household-based sampling strategy of villages in 5 distinct geographical regions (Fig 1B, Anchor Island, North Coast, Bay, South Coast and Channel). The survey included 3501 individuals of all ages ≥6 months residing in 874 households in 19 randomly selected communities. The households were enumerated and geo-positioned and demographic information of the male and female heads of the household collected. Enumeration, but not geopositioning, was achieved for the households in the villages of the South Coast. The timing of the survey was approximately 4 weeks after the peak of the wet season.

Following consent and enrolment from each participant, a short clinical assessment was conducted (including tympanic temperature, history of fever during the previous 48 hours, history of malaria in the last 2 weeks and spleen size in children 2–9 years old) and demographic information collected (age, sex, residency status and history of travel, bed net use). A febrile participant was defined as having a tympanic temperature ≥38.0°C and/or a history of febrile illness in the past 48 hours. Study participants who reported being ill at the time of the survey were diagnosed by RDT (Access Bio, CareStart, USA) and treated if positive with artemether/lumefantrine, as per the national treatment guidelines. Where available the participant’s health records were checked for recent anti-malarial treatment and applicable information recorded.

A 250 μL finger prick blood sample was collected into EDTA-Microtainer tubes (Becton Dickinson, NJ, USA). 50 μL were immediately stabilized in 250 μL RNAProtect (Qiagen, Germany) for RNA studies and stored at ice pack cooling conditions until their transport to a centralized field laboratory. Thick and thin films were prepared for determination of microscopic malaria infection. Haemoglobin measurement was performed with Hemocue HB 301 analyzer. A measurement below 11g/dL was classified as anaemia. Upon return to the centralized laboratory, the RNAProtect fractions were frozen immediately. The remaining 200 μL of whole blood was separated into red blood cells pellets and plasma and promptly frozen.

### LM detection of *Plasmodium spp*. parasites

Giemsa stained blood films were examined under x1000 power. One hundred fields of view were examined before calling a sample “no parasites seen”. When a parasite was observed, counts of both white cells and parasites were commenced, and continued until 300 white cells had been counted. The parasite count was then calculated, based on an assumed white cell count of 8,000 white cells/ μL. However if no further parasites were observed, the process of scanning to a total of 100 fields of view was completed. When only 1 parasite had been observed in 100 fields of view, an assumed count at the notional lower limit of detection of 10 parasites/μL was applied, based on a further assumption of an average of 8 white cells per field of view. All slides were stained within 24 hours at the regional malaria laboratory and read by experienced microscopists, all of whom had completed WHO quality assurance courses. All LM positive slides as well as the slides from all PCR positive / LM negative plus 10% of LM & PCR negative slides were re-read by an Australian Level 1 expert microscopist that was blinded to the PCR results. None of the 10% LM negative slides were found to be positive by the expert microscopist. In case of discrepancies between the two microscopy reads, the read of the expert microscopist was considered final.

### DNA and RNA extraction

Genomic DNA (gDNA) was isolated from red blood cell pellets (100 μL, corresponding to 200 μL whole blood) using FavorPrep 96-well Genomic DNA kit (Favorgen, Taiwan). DNA was eluted in 200 μL elution buffer and stored at -20°C. The RNA isolation procedure from whole blood in RNAProtect cell reagent has been described elsewhere [22], the only exception being an increased elution volume of 60μL of RNase-free water. Due to problems with storage of RNAProtect samples in the field, the quality of the RNA was tested using an RT-qPCR for the human beta globin transcript [23]. This revealed a 10x lower total human RNA concentration than in samples from a comparable study in Papua New Guinea [24]. RNA samples were therefore concentrated 10-fold using a CentriVap Concentrator (Labconco, United States) before testing for the presence of gametocytes.

### Molecular detection of *Plasmodium spp*. parasites

All 3501 DNA samples were first screened using a genus-specific qPCR targeting a conserved region of the 18S rRNA gene [22]. Singleplex species-specific *P*. *falciparum* and *P*. *vivax* Taqman qPCRs and a duplex *P*. *malariae*/*P*. *ovale* qPCR, targeting species-specific regions of 18S rRNA gene, were used to identify species as described previously [22,25]. Prevalence values reported in this study include only those infections confirmed by the species-specific qPCR Taqman assays. Each detection experiment carried a dilution series of plasmids containing the target sequence of each PCR (10^4^, 10^3^, 10^2^, 10^1^, 5, 10^0^ copies/μL), in duplicate, and were used to determine standard curves and therefore estimate parasite densities (reported as 18S rRNA gene copy numbers/μL). All assays were run in 384-well plate format on the Roche LightCycler480 platform. Those infections detected by qPCR, but not by LM, were defined as sub-microscopic infections. *P*. *falciparum* and *P*. *vivax* samples that were positive by species-specific Taqman qPCR were examined for presence of gametocytes using RT-qPCRs targeting the *pfs25* and *pvs25* orthologues, which are expressed only in mature gametocytes, as described previously [22]. All gametocyte assays were also run in 384-well plate format on the Roche LightCycler480 platform.

### *Plasmodium spp*. genetic diversity

All samples that were *P*. *falciparum* or *P*. *vivax* positive were genotyped to determine the multiplicity of infection (MOI) using highly diverse size-polymorphic molecular markers *msp2* for *P*. *falciparum* and *msp1*F3 and MS16 for *P*. *vivax*, respectively. PCR and capillary electrophoresis were performed with slight modifications to the published protocols [26,27]. Genotyping data was analyzed as described previously [26,27].

### Statistical analysis

Study data were collected and managed using REDCap electronic data capture tools hosted at the Walter and Eliza Hall Institute [28]. Analyses were done using the STATA12 statistical software package (College Station, TX). Differences in participant characteristics at enrolment and prevalence differences among geographical areas and groups of individuals were assessed using Chi-square (χ^2^) or Fisher’s exact tests. Differences in median ages and median household size were explored with quantile regression. Univariable and multivariable logistic regression were used for associations of *P*. *vivax* infection and exposure variables. Associations with *P*. *vivax* parasite density were investigated in simple and multivariable linear regression models on only those subjects who tested positive to qPCR diagnosis. Poisson regression analyses were utilized to explore associations between multiplicity of infection and exposure variables.

## Results

### Study population

A total of 3501 Ngella residents across 874 households were surveyed. The gender and age profiles of the participants were representative of the Central Islands Province population, with 52.5% females and a predominance of younger individuals (median age 18 years). The age distribution was as follows: <2 years, 4.7%; 2–4 years, 10.6%; 5–9 years, 14.8%; 10–14 years, 14.3%; 15–19 years, 7.5%; 20–39 years, 27.0%;>40 years, 21.3%. The majority of participants (95.2%) resided in the village for ≥ 2 months. Of 447 participants who spent at least one night outside their village of residence in the last month, 69.1% travelled within Central Islands Province. 73.3% of participants reported having slept under a long lasting insecticidal net the night before and 56.4% owned a bednet for longer than 24 months. Of all households, 84.5% of households reported to have been sprayed with insecticide, and 70.4% of household heads spoke English. Of all participants, 687 (19.4%) had a history of fever in the previous two days, 685 (19.7%) reported feeling unwell/sick at the time of survey and 23.3% had a haemoglobin measurement <11g/dL. No participant aged 2–9 years of age was found to have an enlarged spleen. A detailed description of demographic and clinical characteristics by geographical region is given in S1 Table.

### Prevalence of *Plasmodium* spp. infection by LM

**Fig 2:**
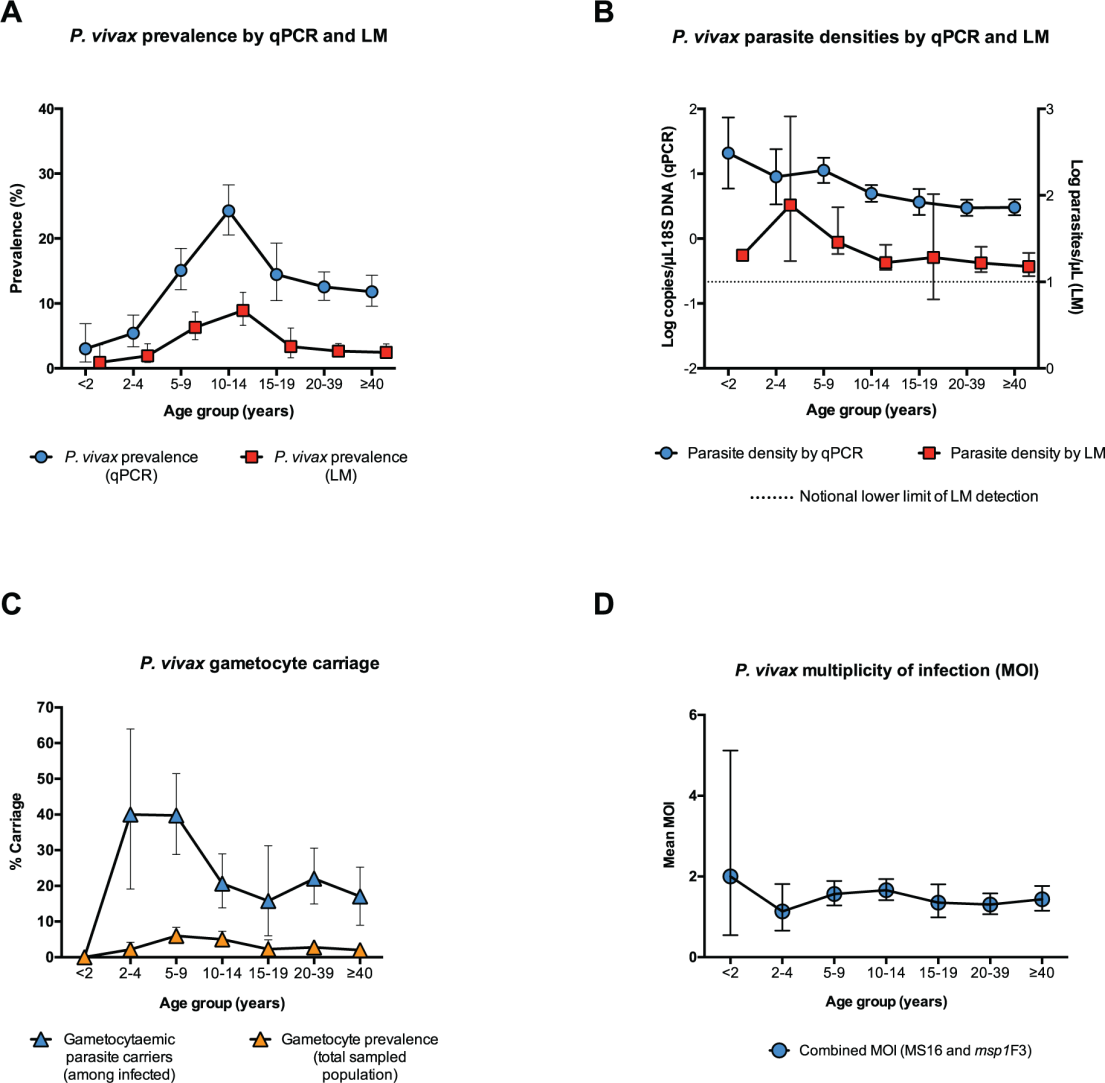
Age trends of *P*. *vivax* infections. A: *P*. *vivax* blood stage parasite prevalence by LM and qPCR (error bars represent binomial 95% confidence intervals, CI_95_). B: *P*. *vivax* parasite densities by qPCR (18S DNA copies/l) and LM counts (parasites/l) (error bars represent 95% confidence intervals, CI_95_). C: Gametocyte prevalence (in the total sampled population) and positivity (only among *P*. *vivax* infected) (error bars represent 95% confidence intervals, CI_95_). D: *P*. *vivax* multiplicity of infection (MOI) of blood stage parasites (error bars represent 95% confidence intervals, CI_95_).

Overall, 130 individuals (3.7%) had *Plasmodium* spp. parasites detectable by LM: 126 *P*. *vivax*, three *P*. *falciparum* mono-infections and one *P*. *vivax/P*. *falciparum* mixed infection. No infections with *P*. *malariae* or *P*. *ovale* were observed. The prevalence of *P*. *vivax* infection varied significantly by geographical region (p<0.001) (Fig 1B) and was lowest in the South Coast and Anchor regions (0.8%), followed by Channel (3.0%) and Bay (3.5%) and North Coast (11.7%). *P*. *vivax* prevalence showed strong age trends and peaked in adolescents 10–15 years of age (8.6%, *p<*0.001) (Fig 2A).

### Prevalence of *P*. *vivax* by qPCR

Overall, 468 participants (13.4%) had qPCR-detectable infections: 463 were *P*. *vivax* mono-infections and five were mixed *P*. *falciparum*/*P*. *vivax* infections (0.14%). The 126 *P*. *vivax* infections and the one mixed infection by LM were confirmed by qPCR. Overall, 72.9% of *P*. *vivax* infections were sub-microscopic. In two of the catchments, Kelarekeha and Vuturua (Fig 1B), only sub-microscopic infections were observed among 59 and 156 individuals surveyed, respectively. *P*. *vivax* qPCR prevalence displayed spatial heterogeneity among the five geographical areas and the 19 catchments, varying from 3.0–38.5%. Prevalence by qPCR was highest in villages on the North Coast (25.0–38.5%) and lowest on Anchor Island (3.0–5.5%) (Fig 1B).

Of 874 households sampled across Ngella, 559 had no infected members, 210 had only one infected member and 105 had two or more infected members. There was no association between household size and probability of being infected (*p =* 0.550). Not taking into account any other variables, there was an increased risk of being infected if at least one other member of the household was infected (OR = 2.59, p<0.001, CI_95_[2.13, 3.16]).

*P*. *vivax* prevalence was age-dependent (*p*<0.001), lowest in<2 years (3.0%, n = 166) and peaking in the 10–14 year old age group (24.3%, n = 499) (Fig 2A). Prevalence of infection did not differ significantly between male and female participants (p>0.650). Participants who were residents (lived in the village ≥ 2 months) were more frequently infected with *P*. *vivax* than non-residents (infected residents: 13.7% vs. infected non-residents 8.3%, *p* = 0.045). Once residency status was taken into account, recent travel (defined as spending at least 1 night away from the village of residence in the last month) was not associated with a difference in infection risk (*p* = 0.300). Those living in a household where the household head speaks English, a proxy for education level, were infected less frequently (12.0%) than those living in a household where the head does not speak English (18.5%, *p<*0.001). There was a moderate increase in risk of *P*. *vivax* infection in those who reported not having slept under a net the night before compared to net users (users: 12.6% vs. non-users: 15.6%, *p =* 0.022).

The majority of *P*. *vivax*-infected individuals (84.6%) neither reported febrile symptoms (defined as history of fever or measured fever at survey) nor feeling ill (85.4%). Six of the 26 participants that had a measured fever at the time of the survey (tympanic temperature ≥38°C, 18.8%) were infected with *P*. *vivax*. Compared to uninfected participants, those with a *P*. *vivax* infection were less likely to report having had febrile symptoms in the previous two days (uninfected 20.0% vs. infected 15.2%, *p =* 0.014) or report feeling unwell at the time of survey (uninfected 20.5% vs. infected 14.6%, *p =* 0.003). A total of 280 *P*. *vivax* infections (60.0%) were both asymptomatic and sub-microscopic. There were no significant differences in the proportion of asymptomatic *P*. *vivax* infections between different age groups (*p*> 0.200) and regions (*p*> 0.240). Of 468 *P*. *vivax*-infected individuals, 19.6% had a haemoglobin<11 g/dL compared to 23.9% of uninfected individuals (*p =* 0.045).

### Multivariable associations with *P*. *vivax* infection

Age was the strongest independent association with *P*. *vivax*. infection, peaking in 10–14 year olds (Table 1, AOR = 8.41, *p<*0.001) and remaining relatively steady for subjects aged 15 years and above (Table 1, 15–19 years AOR = 5.03; 20–39 years AOR = 4.75; ≥ 40 years AOR = 4.40; *p<*0.001). The reference group in this analysis was composed of children aged <2 years. Being a local resident, region of residency and living in a household with at least 1 additional infected member were all associated with an excess risk of infection. Significant protective factors included an English-speaking household head and reporting feeling unwell at the time of the survey. Detailed results are given in Table 1.

**Table 1:** Multivariable associations with *P*. *vivax* infection.

		*n*	PR	AOR	CI_95_	*p*-value^a^
**Region**	Bay	1,161	13.7	**Reference group**		
	Channel	463	13.2	1.01	[0.71, 1.44]	<0.001
	Anchor	857	3.9	0.28	[0.18, 0.44]	
	North Coast	523	31.7	2.52	[1.90, 3.34]	
	South Coast	497	9.9	0.74	[0.52, 1.06]	
**Age group (years)**	<2	166	3	**Reference group**		
	2–4	370	5.4	1.90	[0.68, 5.30]	<0.001
	5–9	517	15.1	5.46	[2.13, 14.01]	
	10–14	499	24.3	8.41	[3.31, 21.38]	
	15–19	263	14.5	5.03	[1.88, 13.40]	
	20–39	940	12.6	4.75	[1.88, 12.02]	
	≥40	746	11.8	4.40	[1.72, 11.23]	
**Head of the household speaks English**	No	661	18.5	**Reference group**		
	Yes	2,465	12	0.76	[0.50, 0.98]	0.030
**Feeling unwell**	No	685	14.3	**Reference group**		
	Yes	2,793	9.9	0.65	[0.48, 0.89]	0.001
**Residency (lived in the village for 2 or more months)**	No	169	8.3	**Reference group**		
	Yes	3,317	13.7	1.91	[1.05, 3.48]	0.004
**Living in a household with at least 1 other *P*. *vivax* carrier**	No	2,235	63.8	**Reference group**		
	Yes	1,266	36.2	1.75	[1.39, 2.20]	<0.001

n = sample size, PR = prevalence of *P*. *vivax* infection, AOR = adjusted odds ratio (predicted difference in odds of *P*. *vivax* infection compared to the baseline odds of *P*. *vivax* infection of the reference group), CI_95_ = 95% confidence intervals.

^a^Overall p-values for variables with multiple levels (age group and region) were derived by use of the likelihood ratio test comparing expanded (includes variable of interest) and nested (does not include variable of interest) models. P-values for the remaining variables are derived from the Wald test.

### *P*. *vivax* parasite densities

Parasite densities by LM count were low (estimated geometric mean 24.3 parasites/μL, CI_95_ [19.2, 30.8]) (Fig 2B). Of 130 LM-detectable infections, 55% (n = 70), were at the assumed limit of practical detection, i.e. approximately 10 parasites/μL. Similarly, *P*. *vivax* parasite densities by qPCR were also low (estimated geometric mean 18S DNA copy numbers of 4.6/μL, CI_95_ [3.9, 5.4]) (Fig 2B). In LM and qPCR positive infections, parasite density by LM (parasites/μL) and by qPCR (18S DNA copies/μL) were correlated (n = 130, R^2^ = 0.76, *p<*0.001). Factors predicting parasite densities by qPCR included age, history of fever in the preceding two days, anaemia (haemoglobin<11 g/dL) and geographical region of sampling. Parasite densities were highest among individuals aged <2 years and decreased in older age groups (*p<*0.001). Detailed results of associations with *P*. *vivax* density are given in Table 2.

**Table 2:** Multivariable associations with *P*. *vivax* density estimates by qPCR.

		n	Geometric mean of parasite density (DNA copies/μl)	Regression Coefficient^§^	CI_95_	*p*-value^a^
**Region**	Bay	1161	2.98	**Reference group**		
	Channel	463	4.73	0.12	[-0.09, 032]	<0.001
	Anchor	857	4.20	0.12	[-0.14, 0.39]	
	North Coast	523	6.21	0.25	[0.09, 0.40]	
	South Coast	497	7.64	0.45	[0.22, 0.67]	
**Age group (years)**	<2	166	20.88	**Reference group**		
	2–4	370	8.99	-0.19	[-0.89, 0.50]	<0.001
	5–9	517	11.28	-0.1	[-0.75, 0.54]	
	10–14	499	4.97	-0.41	[-1.05, 0.24]	
	15–19	263	3.67	-0.52	[-1.19, 0.15]	
	20–39	940	2.99	-0.62	[-1.26, 0.02]	
	≥40	746	3.03	-0.67	[-1.31, -0.02]	
**Fever in the last two days**	No	2798	4.39	**Reference group**		
	Yes	675	6.29	0.2	[0.01, 0.39]	0.036
**Anaemia (haemoglobin<11 g/dL)**	No	2682	4.01	**Reference group**		
	Yes	815	8.46	0.2	[0.03, 0.37]	0.023

^§^The regression coefficients represent the predicted changes in 10-fold parasite density (estimated as 18S DNA copies/μL) compared to the mean parasite density of the reference group.

^a^Overall p-values for variables with multiple levels (age group and region) were derived by use of the likelihood ratio test comparing expanded (i.e. including the variable of interest) and nested (not including the variable of interest) models. P-values for the remaining variables are derived from the Wald test.

### *P*. *vivax* genetic diversity

**Fig 3:**
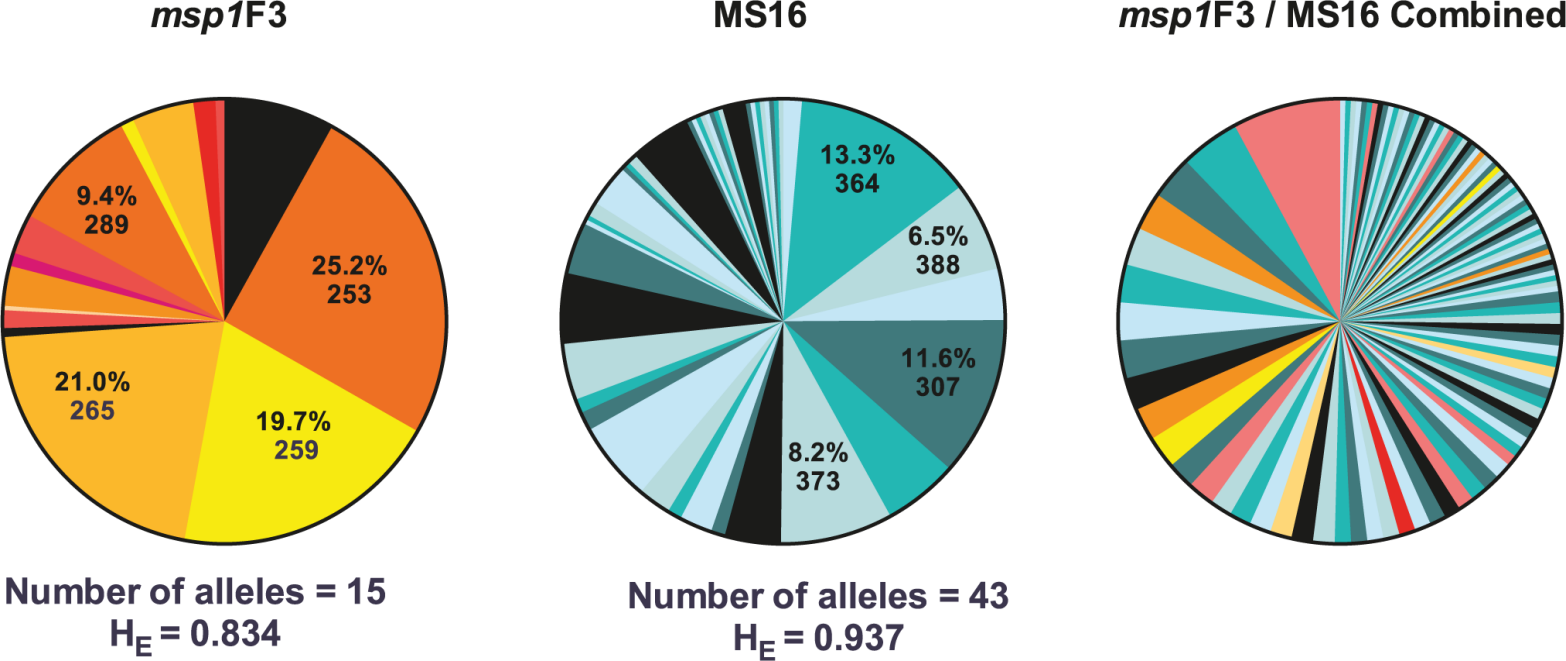
Diversity of *P*. *vivax* genotypic markers *msp1*F3 and MS16. Allelic frequencies of *P*. *vivax msp1*F3, MS16 and the combined *msp1*F3 / MS16 haplotypes. For *msp1*F3 and MS16, the frequency of the four most common alleles and respective sizes (in basepairs) are shown. The number of unique alleles identified and the estimated expected heterozygosity (H_E_) are given below the figure.

Genotyping results for markers *msp1F*3 and/or MS16 were obtained from 349 *P*. *vivax* positive samples. Both markers were highly diverse; 15 *msp1*F3 alleles and 43 MS16 alleles were detected (Fig 3), resulting in an expected heterozygosity (H_E_) of 0.834 for *msp1*F3 and 0.937 for MS16. Out of 349 samples, 102 (29.2%) carried multi-clonal infections. MOI (combined *msp1*F3 and MS16), defined as the concurrent infections per individual, ranged from 1 to 4; mean MOI was 1.36. Mean MOI did not differ significantly among age groups (Fig 2D, *p =* 0.774) or among geographical regions (*p =* 0.610). *P*. *vivax*-infected individuals living in a household with at least one other infected individual had moderately higher mean MOI (1.56) than those who were the sole infected person of the household, but evidence for an association was moderate to weak (mean MOI = 1.42, *p =* 0.086). Mean MOI was positively associated with qPCR parasite density (*p =* 0.007).

### *P*. *vivax* RT-qPCR gametocyte prevalence

In 110 of 468 (23.5%) *P*. *vivax* infections, gametocytes were detected by the *pvs25* RT-qPCR, resulting in a population gametocyte prevalence of 3.14%. More gametocytes were detected in LM-positive (41.5%) than sub-microscopic infections (16.6%, *p<*0.001). The proportion gametocyte positive (defined as percentage of gametocytaemic *P*. *vivax* carriers) was highest in the 2–9 year olds (39.8%, n = 98) and decreasing sharply in the 10–14 year olds (20.7%, n = 121, p>0.001). The lowest proportion gametocyte positive was found among the *P*. *vivax* carriers in the 15–19 years and>40 years age groups (15.8%, n = 38 and 15.9%, n = 88, respectively) (Fig 2C).

### *P*. *falciparum* infection

By qPCR, *P*. *falciparum* was detected in 5 individuals, all of whom were co-infected with *P*. *vivax*. Of these, only four infections were detectable by LM, one as a co-infection with *P*. *vivax* and three as *P*. *falciparum* mono-infections. In two of the LM mono-infections and the mixed infection, only *P*. *falciparum* gametocytes were observed on the blood smear. The range of parasite densities, by qPCR, was 7.35–364 copy numbers/μL and in LM positive samples the densities ranged from 20 to 1430 parasites/μL. The low number of *P*. *falciparum* infections precluded analyses with densities for this species. The presence of gametocytes in the four LM-positive infections was confirmed by *pfs25* RT-qPCR. No gametocytes were detected in the one sub-microscopic *P*. *falciparum* infection by RT-qPCR.

All five *P*. *falciparum*-infected individuals resided in the same village (Halavo, circled in Fig 1B) and ranged in age from 3 to 60 years. The oldest had a history of fever in the preceding two days. Two of the carriers had a haemoglobin measurement <11g/dL. None of the individuals reported to have slept outside the village in the previous month. All five *P*. *falciparum* infections were monoclonal and carried the same *msp2* genotype of the Fc27 subtype.

## Discussion

Solomon Islands has achieved a remarkable 90% reduction in malaria incidence over the last two decades as a result of scaled-up malaria control interventions [6] and is now intensifying its efforts towards malaria elimination [3]. The present study is the first to undertake sensitive molecular diagnosis at this scale in Solomon Islands and the first large epidemiological description of malaria in Ngella since the Malaria Eradication Program (1970–1975). Our findings illustrate a striking distinction between the epidemiology of *P*. *falciparum* and *P*. *vivax* in Ngella.

High prevalence (13.4% by qPCR) and genetic diversity, as well as an increased risk for local residents and evidence of potential within-household transmission indicate considerable levels of endemic *P*. *vivax* transmission. There was significant variation of *P*. *vivax* transmission in different regions of Ngella, with the highest prevalence found on the remote North Coast (25.0–38.5%), which prior to the Malaria Eradication spraying operations was described as holoendemic and having an environment highly favourable to the mosquito [7]. The lowest rates of *P*. *vivax* infection were observed on Anchor (3.9% by qPCR), where 15 years ago a community-based initiative eliminated a substantial number of breeding sites through environmental management [Lodo, personal communication]. It is therefore likely that the presence of suitable larval habitats and vector abundance may be key factors influencing *P*. *vivax* transmission on Ngella.

It remains unclear whether autochthonous *P*. *falciparum* transmission remains in Ngella or parasites are being re-introduced by incoming travelers or returning residents from areas with higher *P*. *falciparum* burden, such Guadalcanal or Malaita provinces. In this survey, only five *P*. *falciparum* cases were identified in the village of Halavo (Fig 1B). As all five infections carried the same *msp*2 allele and four were gametocytaemic, a small local outbreak following recent re-introduction seems more likely. This is reminiscent of the situation in epidemic-prone areas of the Papua New Guinea highlands, where a clonal *P*. *falciparum* epidemic on a background of endemic, low level *P*. *vivax* transmission has been reported [29]. *P*. *falciparum* populations in neighbouring Guadalcanal province were in fact found to be of low genetic diversity [30,31]. Based on case statistics at the local health facilities, 30% of malaria cases detected in Central Islands Province are caused by *P*. *falciparum* [6] indicating that either importation of *P*. *falciparum* parasites is common or that low levels of endemic *P*. *falciparum* transmission may remain in some parts of Ngella. Further studies are therefore required to ascertain the absence of endemic *P*. *falciparum* transmission in this area of Solomon Islands and whether the cases found are the result of inter-island travel.

The current situation of malaria in Ngella (i.e. 3.7% prevalence by LM, clear *P*. *vivax* dominance and absence of enlarged spleens in children 2–9yrs of age) is a consequence of the dramatic reduction in malaria transmission achieved throughout Solomon Islands in the last 20 years [6]. This change is similar to that encountered at the end of 1974, after approximately 5 years of twice-yearly Malaria Eradication Program spraying. Then, prevalence in 2–9 year olds had dropped from pre-spraying rates of 60% to 1.4% and *P*. *vivax* became predominant [7]. Similar shifts in malaria epidemiology were also observed in the elimination provinces of Temotu [9] and Isabel [8]. In Temotu, *P*. *falciparum* accounted for 17.5% of infections in population survey conducted in 2008 [9], but by 2012, the national program’s surveillance system reported only *P*. *vivax* cases from both Temotu and Isabel [6]. This shift in the relative importance of *P*. *falciparum* and *P*. *vivax* are not unique to Solomon Islands and have been reported after periods of sustained malaria control from other settings where *P*. *falciparum* and *P*. *vivax* occur sympatrically, such as the Amazon [21,32], Central America [4] and Thailand [33].

As in other endemic settings [12,13,34], *P*. *vivax* infections were of low density and PCR found three times more infections than LM. The majority of infections were not accompanied by febrile symptoms or anaemia. On the contrary, participants who reported feeling unwell or febrile were less likely to be infected with *P*. *vivax*. While this significantly lower level of febrile symptoms in *P*. *vivax* carriers is likely to be an artifact of the large samples size it does indicate that *P*. *vivax* is not a common cause of fever in Ngella. Whereas asymptomatic *P*. *vivax* infections have been commonly found in areas of high transmission [12,35,36], the advent of molecular diagnosis has revealed that even at low transmission the majority of infections in cross-sectional surveys are symptomless [11,37,38], including in the previous surveys in Temotu [9] and Isabel [8] where 97.1% and 92.9% of *P*. *vivax* infected individuals infections were asymptomatic, respectively.

Both the presence of *P*. *vivax* infections and their level of parasitaemia were found to be strongly age-dependent, albeit in different ways: while *P*. *vivax* parasite densities decreased with age, prevalence of *P*. *vivax* infections rose throughout childhood and only started dropping in adolescents and adults. These contrasting patterns are most likely due to local mosquito biting behavior and acquisition of immunity. *Anopheles farauti*, the only coastal malaria vector in Solomon Islands, is biting predominantly in the early evening (i.e. before 10pm) and outdoors [39], when small children tend to be indoors but older ones still active. The increase in prevalence during childhood is thus likely to represent an increase in exposure to infective bites. At all levels of transmission, immunity to *P*. *vivax* tends to be more rapidly acquired than that to *P*. *falciparum* [40]. Thus, the strong reduction in prevalence and parasite densities with increasing age in Ngella indicate that *P*. *vivax* transmission there remains sufficiently high for relatively rapid acquisition of clinical and anti-parasite immunity.

Despite very low overall parasite densities, gametocytes were detected in almost a quarter of all *P*. *vivax* infections (in 41.5% of LM-positive infections and 16.6% of sub-microscopic infections). Given issues with RNA quality, it is likely that the gametocytaemic reservoir in Ngella was underestimated in our survey and the true prevalence of gametocytes is higher, especially in the sub-microscopic group. Given the rapid and ongoing production of *P*. *vivax* gametocytes, most if not all, blood stage infections could harbor concurrent gametocytes [41]. Whilst sub-patent *P*. *falciparum* infections have been shown to infect up to 43.5% of mosquitoes [17,19], the role of sub-microscopic *P*. *vivax* gametocyte carriage in sustaining transmission is poorly understood. The capacity of sub-microscopic *P*. *vivax* infections to infect mosquitoes has been established in studies from Thailand [18,42,43], Sri Lanka [44], Peru [45] and malaria therapy settings [14,15], but at varying proportions and with weak associations of gametocyte density. Although sub-patent infections may infect fewer mosquitoes, their higher prevalence in endemic settings may mean that the net transmission potential of low-density infections is higher. In Ngella, asymptomatic, sub-microscopic infections of adolescents and adults may thus be an important source of local transmission.

These considerations may constitute a significant challenge to the success of the Solomon Islands malaria control program. The national malaria surveillance system, based on passive case detection and irregular mass blood surveys, only employs traditional microscopy diagnosis. This diagnostic test may not only underestimate the true burden of malaria in the Solomon Islands but also lack the means to detect and attack a substantial part of the *P*. *vivax* transmission reservoir. Despite outstanding gains in the last two decades, traditional tools of the Solomon Islands malaria control program may therefore have reached their effectiveness in the face of a large and silent reservoir of *P*. *vivax* infection.

Our observation that people living in a household with another *P*. *vivax* infected individual is a noteworthy finding. Not only does it indicate likely within-household transmission, but also highlights that reactive case detection strategies [46–48] and focal mass drug administration [34] might be appropriately applied in Solomon Islands. In the Southwest Pacific, MDA campaigns that included primaquine to target the undetectable liver stage parasites have previously been successful in interrupting *P*. *vivax* transmission on Aneytium Island in Vanuatu [49] and Nissan Island in Papua New Guinea [50]. Combining automated registration of observed cases and rapid identification of transmission foci (e.g. in a spatial decision support system) [51] with reactive mass-screen and treat (MSAT) or with focal, household-based mass drug administration [52,53] should therefore be evaluated as possible additional malaria elimination tools in Solomon Islands and neighbouring Vanuatu. All interventions will be most efficacious if they include routine administration of primaquine to all *P*. *vivax* infected individuals. This will however require addressing the challenges posed by the potential primaquine toxicity in G6PD deficient individuals.

## Supporting Information

S1 Checklist STROBE checklist. (DOC)

S1 Table Demographic and clinical characteristics of the study population, by geographical area. (PDF)
