# Supplementary material for: High Rates of Asymptomatic, Sub-microscopic Plasmodium vivax Infection and Disappearing Plasmodium falciparum Malaria in an Area of Low Transmission in Solomon Islands
Source: PLoS Negl Trop Dis. 2015 May 21;9(5):e0003758. doi: 10.1371/journal.pntd.0003758 (PMC4440702; doi:10.1371/journal.pntd.0003758)
Supplement: S1 Checklist — (DOC) [file pntd.0003758.s001.doc]

STROBE Statement—Checklist of items that should be included in reports of ***cross-sectional studies***

|  | Item No | Recommendation |
| --- | --- | --- |
| **Title and abstract** | 1 | (*a*) Indicate the study’s design with a commonly used term in the title or the abstract  **Done in abstract: “a cross-sectional survey of 3501 residents of all ages was conducted in Ngella, Central Islands Province, Solomon Islands.”** |
|  |  | (*b*) Provide in the abstract an informative and balanced summary of what was done and what was found **Done** |
| Introduction | | |
| Background/rationale | 2 | Explain the scientific background and rationale for the investigation being reported  **Addressed in introduction paragraphs 1 and 5.** |
| Objectives | 3 | State specific objectives, including any prespecified hypotheses  **Addressed in the final paragraph of the introduction: “we conducted in May-June 2012 a household-based, cross-sectional survey in Ngella, Central Islands Province to determine how common low-density, asymptomatic infections are in communities where transmission is mesoendemic and whether these infections are gametocyte producers and hence, potential contributors to local transmission.”** |
| Methods | | |
| Study design | 4 | Present key elements of study design early in the paper **The study design was described in detail in the methods section under sub-section “Study population and blood sample collection”.** |
| Setting | 5 | Describe the setting, locations, and relevant dates, including periods of recruitment, exposure, follow-up, and data collection  **The study setting (geography, demography and timeframe) was described in the Methods section, sub-sections “Study Site” and “Study population and blood sample collection”. In addition, a detailed map outlines the location of study communities.** |
| Participants | 6 | (*a*) Give the eligibility criteria, and the sources and methods of selection of participants  **Given in the Methods section, sub-sections “Ethical Statement” (explains enrolment and informed consent processes having recognized the community and cultural values of Solomon Islands) and “Study population and blood sample collection”.** |
| Variables | 7 | Clearly define all outcomes, exposures, predictors, potential confounders, and effect modifiers. Give diagnostic criteria, if applicable  **Demographic and clinical variables of interest detailed in the methods section, sub-section “Study population and blood sample collection”. In the same sub-section, we provided appropriate case definitions (i.e., febrile participant and anaemia). In malaria diagnosis sub-sections (“LM detection of *Plasmodium spp*. parasites” and “Molecular detection of *Plasmodium spp*. parasites”) we have outlined how our diagnostic outcomes were reached (i.e. malaria infection by light microscopy methods and/or by PCR).** |
| Data sources/ measurement | 8* | For each variable of interest, give sources of data and details of methods of assessment (measurement). Describe comparability of assessment methods if there is more than one group.  **Please refer to above (“Variables 7”).** |
| Bias | 9 | Describe any efforts to address potential sources of bias  **In the Methods section, sub-section “DNA and RNA extraction”, we addressed a source of unforeseen technical bias.** |
| Study size | 10 | Explain how the study size was arrived at  **Our overall study size was 3501 participants. This large number of participants was needed for our study to have meaningful statistical power, due to low malaria endemicity in the study area.** |
| Quantitative variables | 11 | Explain how quantitative variables were handled in the analyses. If applicable, describe which groupings were chosen and why  **Described in the “Statistical Analysis” sub-section.** |
| Statistical methods | 12 | (*a*) Describe all statistical methods, including those used to control for confounding **Described in the “Statistical Analysis” sub-section.** |
|  |  | (*b*) Describe any methods used to examine subgroups and interactions |
|  |  | (*c*) Explain how missing data were addressed  **Multivariate analyses done only an participants with complete data** |
|  |  | (*d*) If applicable, describe analytical methods taking account of sampling strategy |
|  |  | (*e*) Describe any sensitivity analyses |
| Results | | |
| Participants | 13* | (a) Report numbers of individuals at each stage of study—eg numbers potentially eligible, examined for eligibility, confirmed eligible, included in the study, completing follow-up, and analysed  **Not applicable, our study is a cross-sectional study, therefore all enrolled participants completed the study.** |
|  |  | (b) Give reasons for non-participation at each stage |
|  |  | (c) Consider use of a flow diagram |
| Descriptive data | 14* | (a) Give characteristics of study participants (eg demographic, clinical, social) and information on exposures and potential confounders  **Demographic and clinical characteristics tabulated (with percent distributions and descriptive statistics) in Supplementary Table 1). Brief descriptions of these data are also provided in the text of the Results section, sub-section “Study Population”.** |
|  |  | (b) Indicate number of participants with missing data for each variable of interest  **Number of participants with missing data and percent distribution of participants represented in each category is provided in Supplementary Table 1.** |
| Outcome data | 15* | Report numbers of outcome events or summary measures **Malaria prevalences were calculated for exposure variables of interest and are provided in the Results section, both in the text (sub-sections “Prevalence of *Plasmodium* spp. infection by LM” and “Prevalence of *P. vivax* by qPCR”) and in Table 1. In addition, malaria frequency (pie) charts, according to geographic origin, are also provided in Figure 1.** |
| Main results | 16 | (*a*) Give unadjusted estimates and, if applicable, confounder-adjusted estimates and their precision (eg, 95% confidence interval). Make clear which confounders were adjusted for and why they were included.  **Univariable logistic regression analysis for malaria infection outcome by qPCR is given in sub-section “Prevalence of *P. vivax* by qPCR”. Multivariable (confounder-adjusted) logistic regression analysis for malaria infection is given in the sub-section “Multivariable associations with *P. vivax* infection” and further detailed in Table 1. The 95% confidence intervals reported in Table 1.**  **Multivariable linear regression (for parasite density analyses on only those subjects who tested positive to qPCR diagnosis) are reported in the sub-section “*P. vivax* parasite densities” and Table 2. The 95% confidence intervals reported in Table 2.**  **Poisson regression (for mean multiplicity of infection analyses) is detailed in the “*P. vivax* genetic diversity” sub-section.** |
|  |  | **(**b) Report category boundaries when continuous variables were categorized **For the age and anaemia categorical variables, the boundaries are provided both in text and table format in the results section (sub-section “Prevalence of *P. vivax* by qPCR” and Tables 1 and 2)** |
|  |  | (*c*) If relevant, consider translating estimates of relative risk into absolute risk for a meaningful time period |
| Other analyses | 17 | Report other analyses done—eg analyses of subgroups and interactions, and sensitivity analyses |
| Discussion | | |
| Key results | 18 | Summarise key results with reference to study objectives **Addressed in discussion paragraphs 1, 5 and 7.** |
| Limitations | 19 | Discuss limitations of the study, taking into account sources of potential bias or imprecision. Discuss both direction and magnitude of any potential bias. **A potential study limitation and recommendations for further investigation relevant to this limitation were discussed in paragraph 3. The technical bias (mentioned here at point 9) was discussed in paragraph 7 of the discussion.** |
| Interpretation | 20 | Give a cautious overall interpretation of results considering objectives, limitations, multiplicity of analyses, results from similar studies, and other relevant evidence **Discussed in the final two paragraphs of the discussion.** |
| Generalisability | 21 | Discuss the generalisability (external validity) of the study results **Placing the study results in the context of the current literature of the field done in discussion paragraphs 2, 3, 4 and 5.** |
| Other information | | |
| Funding | 22 | Give the source of funding and the role of the funders for the present study and, if applicable, for the original study on which the present article is based **Provided under the “Funding Statement” sub-section.** |

*Give information separately for exposed and unexposed groups.

**Note:** An Explanation and Elaboration article discusses each checklist item and gives methodological background and published examples of transparent reporting. The STROBE checklist is best used in conjunction with this article (freely available on the Web sites of PLoS Medicine at http://www.plosmedicine.org/, Annals of Internal Medicine at http://www.annals.org/, and Epidemiology at http://www.epidem.com/). Information on the STROBE Initiative is available at www.strobe-statement.org.
