# Supplementary material for: High Rates of Asymptomatic, Sub-microscopic Plasmodium vivax Infection and Disappearing Plasmodium falciparum Malaria in an Area of Low Transmission in Solomon Islands
Source: PLoS Negl Trop Dis. 2015 May 21;9(5):e0003758. doi: 10.1371/journal.pntd.0003758 (PMC4440702; doi:10.1371/journal.pntd.0003758)
Supplement: S1 Table — (PDF) [file pntd.0003758.s002.pdf]

S1 Table Demographic and clinical characteristics of the study population, by geographical area.

Study Population Characteristic	Total	Anchor	Bay	Channel	North Coast	South Coast	*p*-value
Number of participants sampled	3501	857	1161	463	523	497	<0.001
Number of households sampled	874	202	306	101	133	132	<0.001
**Demographic characteristics**							
Median household size	6	6	6	6	6	5	1.000
Median age (years)	18	20	16	17	18	20	0.066
Female %	52.5	49.8	52.9	54.4	55.45	51.3	0.255
Residents %	95.2	95.8	96.1	91.1	93.86	97	<0.001
IRS %	84.5	89.5	83.2	75.2	84.94	87.2	<0.001
Head of household formal education %	89.6	93	87.3	89.1	86.68	92.7	<0.001
Head of household speaks English %	70.4	85.5	82.7	73.6	66	78.5	<0.001
Spent the night away in previous month %	12.9	16.7	8.9	14.3	15.12	11.8	<0.001
Within CIP travel %	69.1	78.8	69.4	76.6	50.67	59.1	
Guadalcanal travel %	29.6	20.3	29.5	23.4	46.67	40.9	<0.001
Travel to other provinces %	1.3	0.8	2.1	0	2.66	0	
**Clinical Characteristics**							
History of malaria in previous 2 weeks %	3	1.9	3.5	3.2	4.4	1.8	0.031
Taken antimalarials in previous 2 months %	5.3	2.9	6.47	7.4	5.6	4.9	0.003
Slept under a LLIN the previous night %	73.3	73.5	71.7	64.2	77.5	80.5	<0.001
LLIN usage duration %							
≤ 6 months	0.7	0.9	1.1	0.4	0.2	0.4	
≤ 12 months	12.4	13.8	8.8	6.3	27.7	7.8	<0.001
≥ 24 months	56.4	55	61.2	57.7	33.1	71.2	
Feeling unwell %	19.7	16.6	18.5	25.1	26.1	16.1	<0.001
History of fever in previous 2 days %	19.4	15.4	17.7	24.6	27.8	16.6	<0.001
Measured fever (tympanic ≥38°C) %	0.9	1.9	0.6	0.4	1.2	0.2	0.01
Anaemia <11g/dl %	23.3	19.7	26.3	23.6	20.9	25	0.006

Not all participant responses were recorded for each parameter in the questionnaire, therefore percentages were calculated based on the number of available answers.
